# A leucine aminopeptidase is involved in kinetoplast DNA segregation in *Trypanosoma brucei*

**DOI:** 10.1371/journal.ppat.1006310

**Published:** 2017-04-07

**Authors:** Priscila Peña-Diaz, Marie Vancová, Christian Resl, Mark C. Field, Julius Lukeš

**Affiliations:** 1 Institute of Parasitology, Biology Centre, Czech Academy of Sciences, České Budějovice (Budweis), Czech Republic; 2 Faculty of Science, University of South Bohemia, České Budějovice (Budweis), Czech Republic; 3 School of Life Sciences, University of Dundee, Dundee, United Kingdom; 4 Canadian Institute for Advanced Research, Toronto, ON, Canada; New Jersey Medical School, UNITED STATES

## Abstract

The kinetoplast (k), the uniquely packaged mitochondrial DNA of trypanosomatid protists is formed by a catenated network of minicircles and maxicircles that divide and segregate once each cell cycle. Although many proteins involved in kDNA replication and segregation are now known, several key steps in the replication mechanism remain uncharacterized at the molecular level, one of which is the nabelschnur or umbilicus, a prominent structure which in the mammalian parasite *Trypanosoma brucei* connects the daughter kDNA networks prior to their segregation. Here we characterize an M17 family leucyl aminopeptidase metalloprotease, termed TbLAP1, which specifically localizes to the kDNA disk and the nabelschur and represents the first described protein found in this structure. We show that TbLAP1 is required for correct segregation of kDNA, with knockdown resulting in delayed cytokinesis and ectopic expression leading to kDNA loss and decreased cell proliferation. We propose that TbLAP1 is required for efficient kDNA division and specifically participates in the separation of daughter kDNA networks.

## Author summary

Trypanosomes bear a single mitochondrion with its genome (kinetoplast, or kDNA) arranged as a network of circular molecules. kDNA is essential for the *Trypanosoma brucei* life cycle, as it is required for proper functioning of the mitochondrion and progression through the insect vector. Division of kDNA is synchronized with the replication of nuclear DNA and additional cell cycle events. Though the mechanism of kDNA replication is well understood and proteins mediating this process identified, several key steps, including segregation, remain poorly known, in part due to the absence of characterized proteins specifically functioning at this stage. Here we report leucine aminopeptidase 1 (LAP1) as the first identified component of the nabelschnur, or umbilicus, a structure observed during the separation of daughter kDNA networks, whose expression is required for correct kinetoplast segregation.

## Introduction

Kinetoplast (k) DNA is one of the hallmarks of the family Kinetoplastea, and is a unique form of mitochondrial (mt) DNA. Kinetoplastids are a species-rich group, which includes numerous obligatory parasitic trypanosomatids, such as *Trypanosoma brucei*, *T*. *cruzi* and *Leishmania* spp., causative agents of serious human diseases. In all known trypanosomatids, kDNA exists as a single network composed of mutually catenated circular molecules of two categories–maxicircles and minicircles—that are extended and densely packaged into a highly organized discoid structure [1]. The kDNA network is located in the mt lumen adjacent to the basal body of the single flagellum, to which it is physically attached via the tripartite attachment complex (TAC). The TAC is a structure comprised of both exclusion zone and unilateral filaments, which are assembled with a region of non-corrugated inner mt membrane between them [2]. Unlike the mt DNA of most eukaryotes, kDNA divides once per cell cycle. Whilst this occurs immediately prior to nuclear DNA synthesis, replication of kDNA is in synchrony with nuclear division [3]. In *T*. *brucei*, kDNA is essential for mt functions, although recently specific mutations have been described that can facilitate viability without kDNA [4]. However, this is not the case for most infective strains found in the field, as treatment with ethidium bromide, which leads to kDNA loss, proves effective in controlling the spread of the parasite among cattle [5].

In the African trypanosome *T*. *brucei*, kDNA is composed of ~25 maxicircles, each 23 kb long, and ∼ 5,000 minicircles that are ~1 kb in size [6]. All maxicircles have essentially identical sequence and represent homologues of typical mtDNA. Maxicircles contain 18 protein-coding genes, the products of which are mostly subunits of respiratory complexes plus two ribosomal RNAs [6,7]. Most of these genes are present in an encrypted form and in order to become translatable, their transcripts require extensive post-transcriptional insertions and deletions of uridines *via* RNA editing [8–10]. Minicircles are very heterogeneous in sequence and encode hundreds of different guide (g) RNAs (usually one per minicircle) that provide sequence information for editing the maxicircle transcripts [10,11]. Maxicircles and minicircles probably constitute distinct networks that are mutually interlocked within the single kDNA disk [12].

A unique feature of the trypanosomatid kDNA is that its constituents are non-supercoiled open circles, catenated with three neighbors *via* a single interlock, creating a chainmail-like structure [1,13,14]. Within this catenated kDNA disk the circles remain closed throughout the cell cycle and only during replication are they decatenated by the action of topoisomerase(s) II, and transported into a region between the kDNA disk and the basal body termed the kinetoflagellar zone [15,16]. Decatenated minicircles then undergo replication via a θ structure. The replication products of each and every minicircle, distinguished from their pre-replicated neighbors by retaining nicks and gaps, are re-attached into the kDNA disk within two antipodal sites, upon which the strand discontinuities are sealed [17,18]. Therefore, for every detached and replicated minicircle, two minicircles re-attach, doubling the size into a catenane of about 10,000 minicircles. In *T*. *brucei*, progeny minicircles accumulate on opposite sites of the disc at the antipodal sites [15]. Subsequently, the network divides into two by an unknown process requiring at least one of three mt DNA polymerase [19,20] and a DNA ligase that closes the nicks [21]. Maxicircle replication occurs within the kDNA network, and many proteins, including multiple helicases and three mt DNA polymerases are also required for this process [18,20,22].

In *T*. *brucei* the accumulation of replicated minicircles at the antipodal sites results in the formation of a dumbbell-shaped network, whereas in other trypanosomatids, such as *Crithidia fasciculata*, *T*. *cruzi*, *Leishmania* and *Phytomonas* spp., minicircles are uniformly distributed around the periphery of the kDNA disk, forming a peripheral ring [23–25]. To explain such distinct replication mechanisms, it has been proposed that in the latter group the kDNA disk rotates, distributing the re-attaching minicircles around the periphery of the kDNA network [26]. In *T*. *brucei*, however, the disk remains stationary, and the replicated network divides by an unknown mechanism. It is assumed that this mechanism is unique to *T*. *brucei* and its close relatives due to the particular nature of the kDNA segregation process present in this species.

Situated in the posterior region of the cell, the kDNA disk is physically linked to the basal and pro-basal bodies by the tripartite attachment complex (TAC) [2]. It has been postulated that, at the moment of cell division, the basal bodies align and direct kDNA segregation, a process orchestrated via the TAC [27]. The physical separation of the progeny kDNA networks as observed by electron microscopy, has been associated with the formation of a filament-resembling structure termed the nabelschnur or umbilicus [28]. This structure, so far observed exclusively in *T*. *brucei*, constitutes the final physical connection between the newly replicated daughter kDNA networks [28,29]. Although the kDNA of *T*. *brucei* is one of the best-studied mt genomes, the mechanism(s) governing this highly precise division remain largely unknown.

Here we characterize the function of a leucyl aminopeptidase, TbLAP1, in the segregation of dividing kDNA. LAPs are homohexameric metallopeptidases classified into either the M1 or M17 protease families [30] that cleave N-terminal amino acids from proteins, particularly, but not exclusively, L-leucine. Members of the M1 family carry a canonical HEXXH motif in their active site, whereas the M17 family members lack this motif and require two metal ions per monomer for activity [31]. LAPs have diverse subcellular localizations; initially found in the cytosol, they have been subsequently encountered in chloroplasts [32], on bacterial surfaces [33], or teguments of parasitic helminths [34]. Moreover, in *Escherichia coli* LAPs bind DNA [35], while they are amongst secreted proteins in other bacteria such as *Mycoplasma* [33]. Despite high sequence conservation, members of the M17 protease family perform a range of moonlighting functions in diverse organisms. Amongst these functions, LAPs regulate meiosis in fungi [36], are involved in infectivity of various bacteria, yeast and parasitic protists [37–39], regulate stress responses and signal transduction [40], act as molecular chaperones that protect proteins from heat inactivation and assist in their refolding in plants [41] and finally, are required for glutathione metabolism and recycling [42]. Regardless of these diverse roles of LAPs, the exact mechanisms of their many moonlighting functions remain to be clarified.

**Fig 1:**
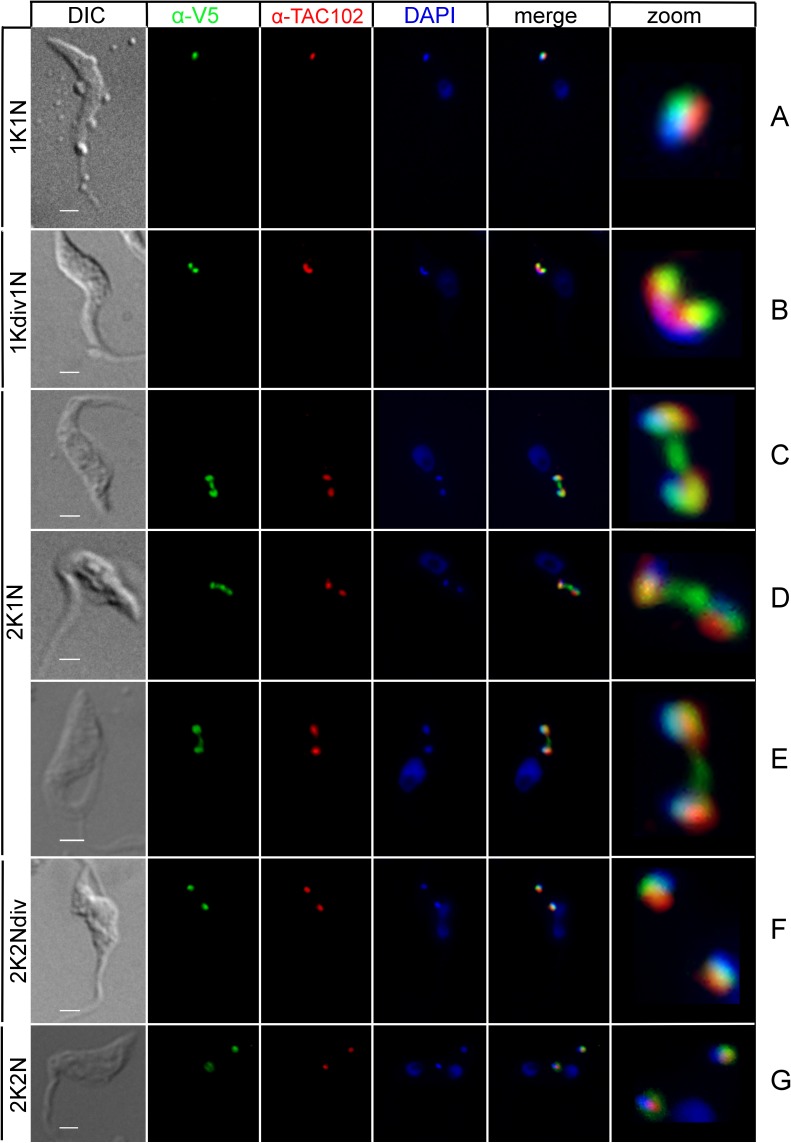
Immunofluorescence of procyclic *T*. *brucei* expressing *in situ*-tagged TbLAP1-V5 protein. Cells were double labeled with polyclonal anti-V5 (green) and anti-TAC102 (red) antibodies (see Materials & Methods for details). DAPI (blue) shows the location of the nucleus and kDNA. Zoom shows enlargement of the kDNA and associated region from the merge. The V5 signal co-localizes with the kDNA disk but does not co-localize with the anti-TAC102 antibody (A). Once kDNA division has been completed (the “dumbbell” kDNA shape), TbLAP1 appears in two foci, one at each tip of the structure, meanwhile TAC102 is found in an elongated form close to segregation (B). Once kDNA segregation is initiated, the TbLAP1 signal extends into the nabelschnur, and at the same time co-localizes with the kDNA disk (C). As the segregation process continues, the nabelschnur elongates, and concentration of TbLAP1 at the nabelschur centre increases along with the protein located in kDNA (D). kDNA segregation is completed following fading of the nabelschnur, with the remaining TbLAP1 signal co-localized with kDNA (E). The TAC signal has clearly divided into two structures and co-migrates with the kDNA/TbLAP1 association (F). 2K2N cells display similar localisations for both proteins as do the 1K1N type (G). Scale bars: 1 μm.

In this paper we report that TbLAP1 is a mt protein that dynamically associates with kDNA during the cell cycle (Fig 1). The protein localizes to kDNA and the nabelschnur, the proteinaceous link connecting progeny kDNAs at late stages of segregation, indicating a role of TbLAP1 in resolving kDNA replication.

## Results

### Subcellular localization of TbLAP1

TbLAP1 (Tb927.8.3060) is a predicted protein of ~70 kDa. Several algorithms (PSORTII [43], iPSORT [44], MitoProt II [45] and TargetP 1.1 [46]) predict mt localization for TbLAP1 based on the presence of an N-terminal mt targeting signal. Due to this we chose to investigate TbLAP1 further. Indeed, TbLAP1, *in situ* tagged at the C-terminus with either green fluorescent protein (GFP; S6 Fig) or V5 was throughout the cell cycle exclusively associated with the kDNA disk (Fig 1).

Significantly, TbLAP1 distribution within the kDNA undergoes substantial changes during cell division. During the G_1_ phase, TbLAP1 co-localizes with the kDNA but not with TAC102, a component of the TAC, a filamentous structure that connects kDNA with both the basal body and the flagellum (Fig 1). The onset of mitosis in *T*. *brucei* is marked by basal body division followed by duplication of the TAC. Replicating in parallel with the TAC, kDNA initially forms a dumbbell-like structure, where TbLAP1 bifurcates into two lobes that bind each side of the divided but as yet unsegregated kDNA disk (Fig 1B). Shortly afterwards, the kDNA disk commences segregation into two daughter networks, positioned perpendicular to one another (Fig 1C, 1D and 1E). At this stage, the characteristic nabelschnur becomes apparent between the newly replicated kDNA networks [28,29]. As the daughter kDNA discs progressively separate, the nabelschnur extends with a small, yet prominent, TbLAP1 focus at its center (Fig 1C and 1D). Once the daughter kDNA disks have completed their realignment, the TbLAP1 signal within the nabelschnur decreases and eventually remains confined to a spot overlaying each newly synthesized kDNA disk (Fig 1G). It is important to highlight that TbLAP1 localization overlaps with kDNA, but is clearly distinct.

**Fig 2:**
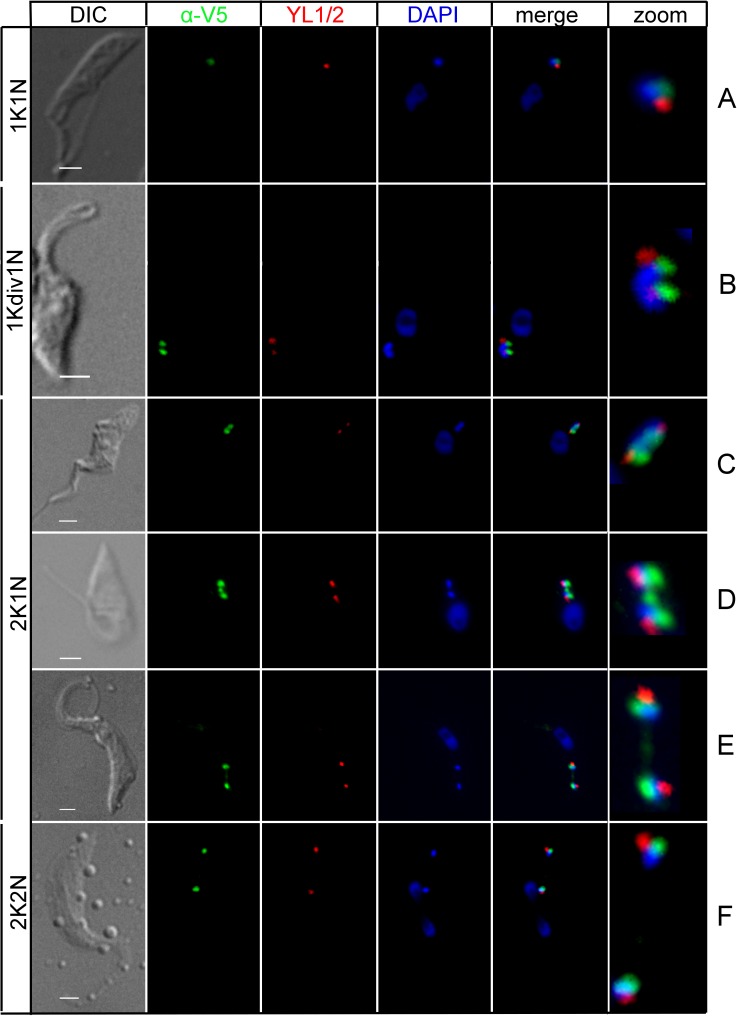
Immunofluorescence assay of cells expressing *in situ*-tagged TbLAP1-V5. Cells were double-labeled with polyclonal anti-V5 (green) and YL1/2 (red) antibodies. DAPI (blue) shows the location of the nucleus and kDNA. Zoom shows enlargement of kDNA and associated region from merge. TbLAP1 (detected with anti-V5 antibody) co-localizes with kDNA, which is positioned next to the basal body (A). TbLAP1 forms two foci overlapping with the dividing kDNA; the basal body has also divided (B). As kDNA begins segregation, the signal of the TbLAP1 arranges in a bi-lobular, dumbbell-like structure (C). The nabelschnur becomes apparent at the start of progeny kDNA segregation (D). At an advanced stage of kDNA segregation, the nabelschnur begins to fade, with only a small accumulation of TbLAP1 evident in the center of the fading link (E). Once the kDNAs have segregated, the nabelschnur completely disappears and the TbLAP1 signal remains localized exclusively to the kDNA disks, with the basal body juxtaposed next to it (F). Scale bars: 1 μm.

The division of the basal body was also followed as a landmark for the dynamic localization of TbLAP1 during the division of procyclic *T*. *brucei*. This structure has been shown to divide prior to, and promote segregation of the daughter kDNA disks [47]. Immunolocalization of the YL1/2 epitope, specific for mature basal bodies, does not overlap with that of TbLAP1 (Fig 2), demonstrating that these structures indeed segregate prior to the separation of the kDNA disks (Fig 2B and 2C). Staining of the basal bodies depicts localization of TbLAP1 to kDNA upon division and its dynamic localization along the duplicating disk (Fig 2C). The TbLAP1 signal in Fig 2D and 2E indicates that the nabelschnur emerges coincident with immunostaining of anti-TAC102 antibody (Fig 1). After segregation of these newly divided disks, the basal bodies align perpendicularly, standing at 180° angle to each other, as both kDNAs separate and align for cytokinesis (Fig 2E and 2F).

**Fig 3:**
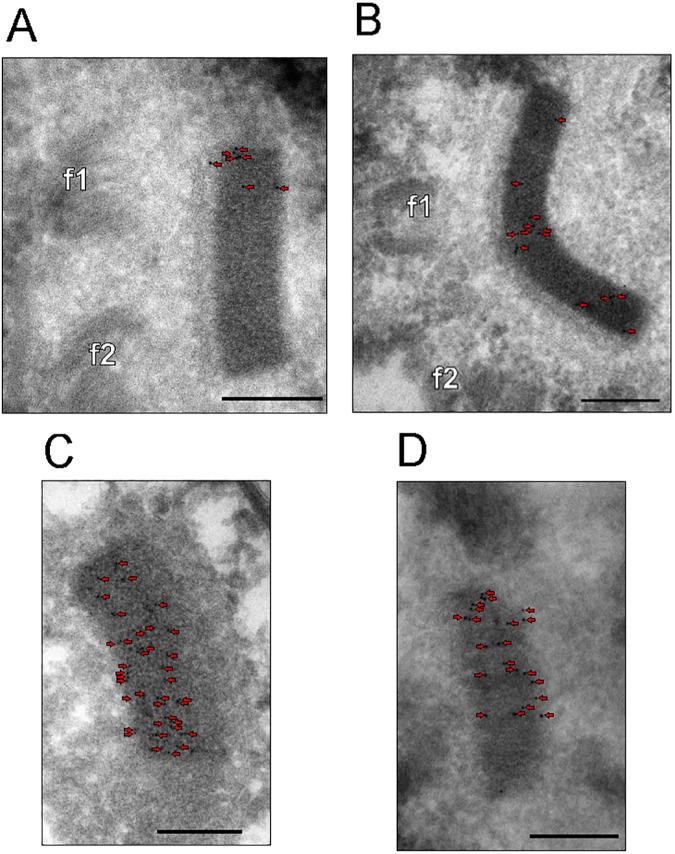
Immunolocalization of *in situ* tagged TbLAP1-V5 protein by cryo-transmission electron microscopy. Representative kDNA disks displaying distributions of gold nanoparticles either in clusters (A, B) or with random distribution (C,D), as evaluated by Ripley’s function. f1, f2 –basal bodies of the dividing flagella. Scale bars: 200 nm. (n = 342, S1 Table)

Localization of *in situ* V5-tagged TbLAP1 into the kDNA disk was further confirmed by cryosectioned transmission electron microscopy *via* immunodecoration with colloidal gold-labeled anti-V5 antibodies, with abundant gold particles found exclusively on the electron-dense kDNA disk. Unlike with immunofluorescence, except for the “dumbbell-shaped” kDNA, it is not possible to assess at which stage of cell division these kDNAs are, but based on their sizes these are most likely interphase networks (Fig 3). The spatial distribution of the signal in the observed kDNAs was evaluated by Ripley’s K function [48,49]. The statistical analysis indicates that the anti-V5 antibody signal exists in two modes and either forms clusters (Fig 3A and 3B), or is randomly distributed throughout the kDNA (Fig 3C and 3D). In the analyzed samples (n = 20), 40% of the gold particles found display cluster formation, while the remaining 60% are randomly distributed (n = 342; S1 Table).

### Knockdown of TbLAP1 by RNAi leads to accumulation of 2K2N cells

**Fig 4:**
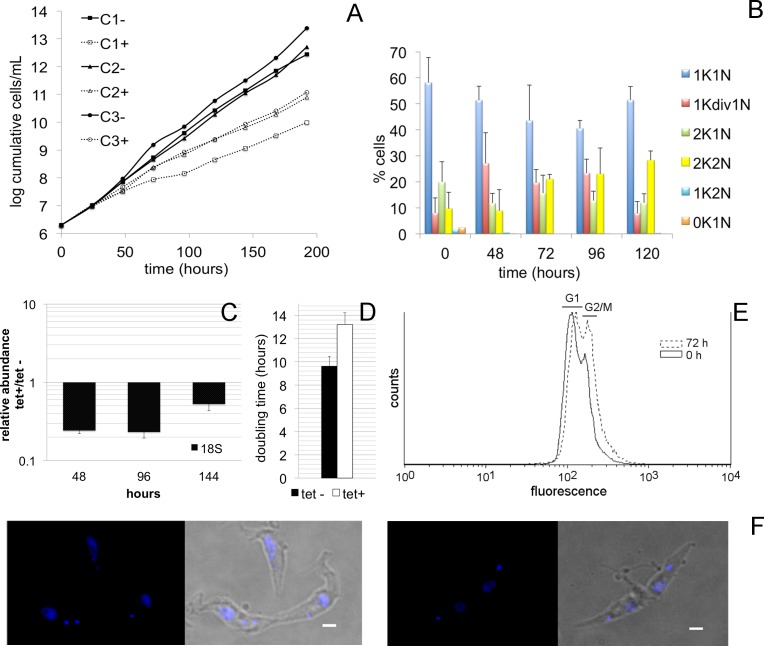
RNAi-mediated down-regulation of TbLAP1 results in a growth defect. (A) Growth of trypanosomes in the presence (white, dashed lines) and absence (black, unbroken lines) of tetracycline to induce TbLAP1 RNAi silencing, over an 8-day period. Three clones were used for the analysis. Cultures were diluted to 2 x 10^6^ cells/mL and cell number was measured every 24 hrs. (B) DAPI counts of TbLAP1 RNAi induced and uninduced cells. Two hundred cells per time point were counted and used for the analysis. (C) Quantitative real-time PCR analysis of uninduced and induced TbLAP1 RNAi cell lines normalized to 18S rRNA. Three time points of induction (48, 96, 144 hrs) were used for the analysis. (D) Average doubling time of the uninduced and induced RNAi cell line. (E) PI staining analysis of uninduced and induced TbLAP1 RNAi cell lines. The overall PI fluorescence did not change significantly between uninduced (0 hrs) and induced (72 hrs) RNAi cell lines, but a shift in populations was observed when uninduced and induced were overlaid. (F) Bright field and DAPI stained images, exhibiting the representative 2K2N cells observed in culture.

RNAi-mediated down-regulation of the TbLAP1 protein induces a growth defect (Fig 4A and 4D), which is nonlethal but causes a delay in cytokinesis. Real-time qPCR analysis confirmed the TbLAP1 transcript is reduced by 80% at 48 and 96 hrs post-induction, but seems to increase at 144 hrs to 50% when normalized to 18S rRNA levels (Fig 4C). At this time a substantial proportion of 2K2N cells was observed (Fig 4B). Propidium iodide (PI) staining by FACS analysis indicated that the variability on the fluorescence of this compound associated with the cell population was greater for the RNAi-induced cells than the uninduced cells. Nevertheless, overall fluorescence does not vary between induced and uninduced cell lines, an indication that DNA content is not significantly changed upon silencing of TbLAP1 (Fig 4E). The actual evidence is that cell duplets accumulate in culture, represented by 2K2N cells, in accordance with the DAPI staining results. RNAi-ablated cells display a dividing kDNA, prior to cytokinesis (Fig 4F).

### Effects of inducible ectopic expression of TbLAP1-HA

**Fig 5:**
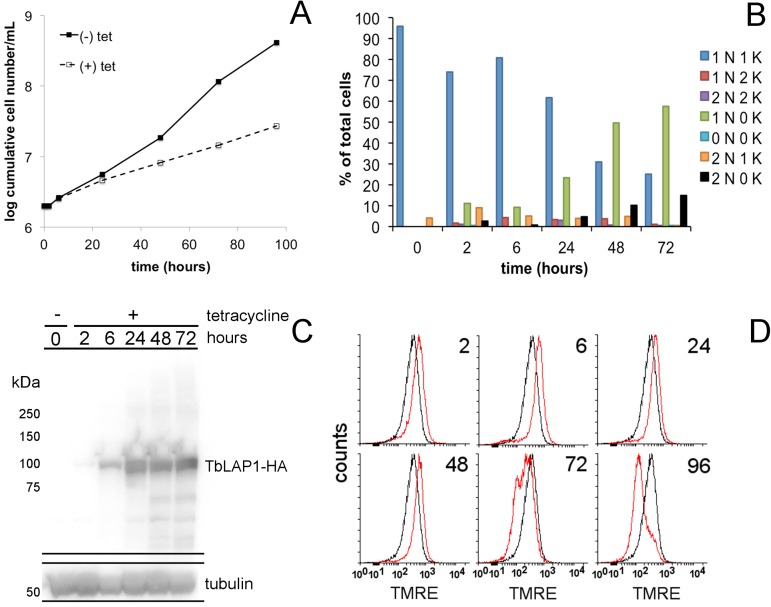
Characterization of procyclic *T*. *brucei* expressing ectopically TbLAP1-HA. (A) Growth of trypanosomes in SDM-79 in the presence (dotted line) and absence (continuous line) of tetracycline (Tet). (B) DAPI counts in induced cells with Tet for ectopic expression of TbLAP1-HA. Two hundred cells per time point were counted and used for the analysis. (C) Western blot analysis of ectopically expressed TbLAP1-HA using monoclonal anti-tubulin antibody as a loading control. 5 x 10^6^ cells per sample were loaded per well. (D) Mitochondrial membrane potential measurement using TMRE of uninduced (black line) and induced (red line) cells expressing TbLAP1-HA (2, 6, 24, 72 and 96 hrs of induction). The measurement is a representative of an assay performed in three independent experiments.

Ectopic expression of TbLAP1-HA in procyclic *T*. *brucei* induced a significant growth defect (Fig 5A), which was manifested by the accumulation of 0K1N cells (Fig 5B) and a defect in mt membrane potential (Fig 5D). Ectopic expression of TbLAP1-HA was monitored by Western blot using anti-HA antibody (Fig 5C). A concomitant loss of kDNA was observed 2 hrs post-induction, as shown by the significantly increased proportion of 0K1N cells, almost complete disappearance of 2K2N cells after 48 hrs, and the increase of 0K2N cells after 72 hrs (Fig 5B).

The staining of procyclic trypanosomes expressing TbLAP1-HA with MitoTracker resulted in an uneven, patchy distribution of the probe throughout the reticulated mitochondrion (S8 Fig), suggesting a defect in mt membrane potential. Indeed, we observed a very rapid effect on this parameter, evident after only 2 hrs of induction of TbLAP1-HA. Over-polarization of the mt membrane continued until 72 hrs of induction, after which the mt membrane potential decreased and fell below values in uninduced cells, presumably due to failure of the mt membrane and/or loss of viability (Fig 5D).

**Fig 6:**
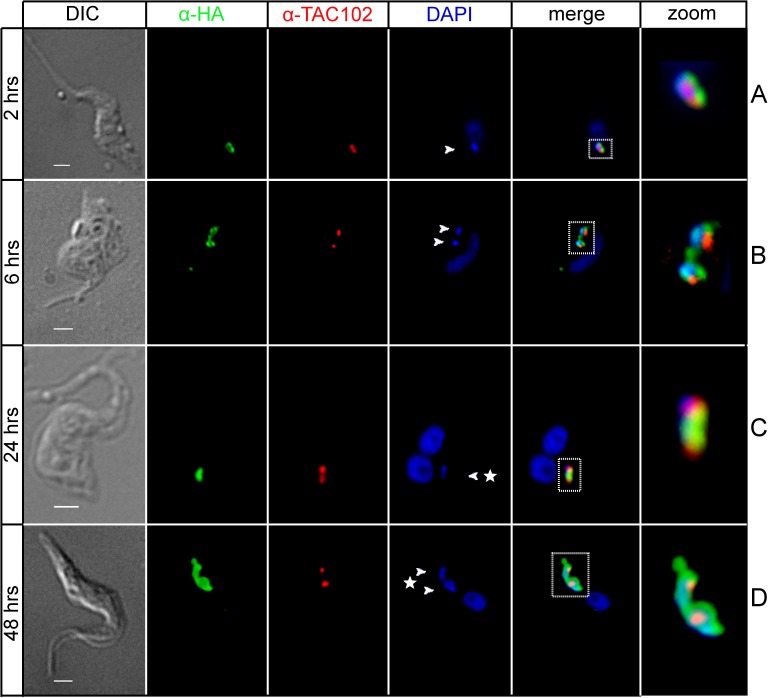
Immunofluorescence of cells ectopically expressing the TbLAP1-HA protein, double labeled with polyclonal anti-HA (green) and anti-TAC102 antibodies (red). The latter antibody visualizes the dynamics of the tripartite attachment complex (TAC). DAPI (blue) shows the location of the nucleus and kDNA; arrowheads and asterisks denote aberrant kDNA. Squares denote area displayed as zoom. B) After 2 hrs (A) and 6 hrs of ectopic expression of TbLAP1-HA (B), the apparition of 1K2Ndiv cells (not yet segregated cells with kDNA in division) became evident as depicted by two cells failing to segregate their already divided kDNAs; note the structure of the nabelschnur holding the kDNAs and TAC together at the posterior end of the cell. Division of TAC and nucleus occurred, as shown by two TAC102 signals and two nuclei, without prior segregation of the TAC structures and the kDNAs, which remained at the posterior end of the cell (C). Representative cell displaying accumulation of TbLAP1-HA around aberrantly sized kDNAs (D). Scale bars: 1 μm.

**Fig 7:**
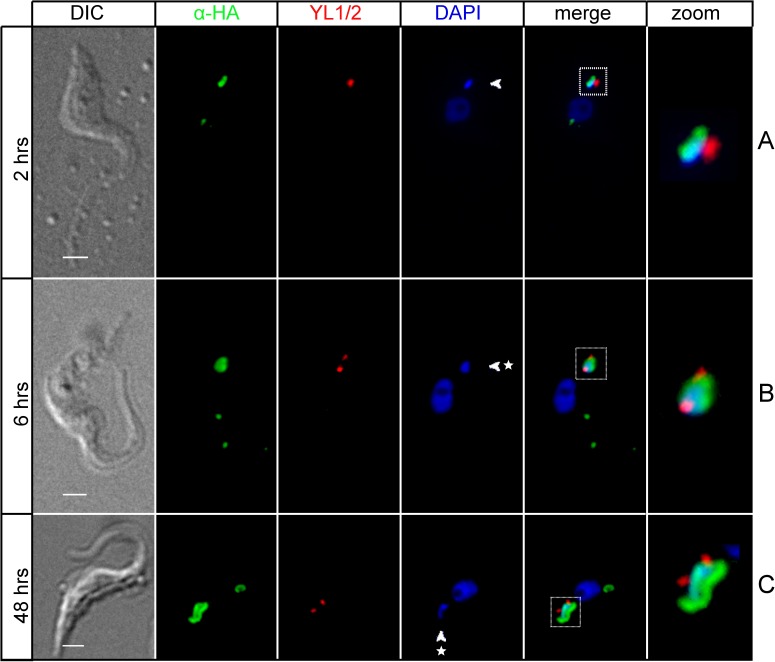
Immunofluorescence of cells ectopically expressing TbLAP1-HA protein, double labeled with polyclonal anti-HA (green) and YL1/2 antibodies (red). The latter antibody visualizes the dynamics of the basal body. DAPI (blue) shows the location of the nucleus and kDNA; arrowheads and asterisks denote aberrant kDNA. Squares denote areas displayed in zoom (approximately 1μm). TbLAP1-HA accumulates around the undivided kDNA (A). Basal body divides and initiates segregation, regardless of the accumulation of TbLAP1-HA around kDNA (B and C). Scale bars: 1 μm.

**Fig 8:**
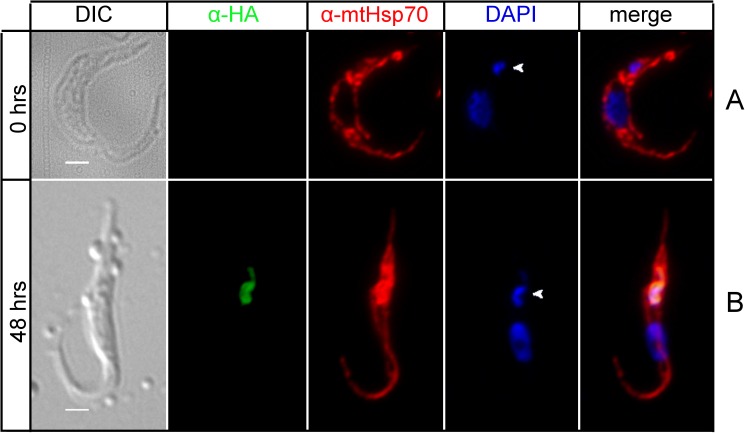
Immunofluorescence of cells ectopically expressing the TbLAP1-HA protein, double labeled with polyclonal anti-HA (green) and monoclonal anti-mtHsp70 antibodies (red). DAPI (blue) shows the location of the nucleus and kDNA. Arrows indicate kDNA. Uninduced cells (A) display mitochondrial staining. 48 hrs post-induction (B) cells display accumulation of mtHsp70 around the TbLAP1-HA signal; the reticulated structure of the mitochondrion is less evident along with the accumulation of mtHsp70. Scale bars: 1 μm.

When TbLAP1-HA expressing trypanosomes were immunodecorated with anti-TAC102 antibody, TAC segregation was heavily affected by the halt in kDNA separation (Fig 6). Cells expressing TbLAP1-HA are able to duplicate their kDNA, but proper separation fails (Fig 6B, 6C and 6D). Aberrant kDNA segregation was observed as early as 6 hrs post-induction (Fig 6B). Neither the division of the basal body, nor its segregation were significantly affected (Fig 7). Immunodecoration of the same cells with anti-mtHsp70 antibody, a marker for the mt matrix [50], demonstrated accumulation of mtHsp70 around the kDNA disk after 48 hrs of induction, with a concomitant loss of the reticulated network structure of the mitochondrion (Fig 8B). Expression of TbLAP1-HA was associated with extensive kDNA loss that peaked after 72 hrs of induction, when over 60% of cells became akinetoplastic (Fig 8A) and lost the reticulated mt network, as well as the focal distribution of TbLAP1-HA (Fig 8B).

## Discussion

Here, we report the first case of a mitochondrion-localized LAP, namely TbLAP1 in *T*. *brucei*, which has a unique and unexpected function. In this compartment, TbLAP1 is present in both the kDNA disk and the nabelschnur, where it undergoes dynamic relocations during kDNA division and segregation. Dynamic repositioning during the kDNA replication is known for a number of kDNA-associated proteins, such as the one observed for DNA polymerase ID [1,6,9,51,52], yet to the best of our knowledge TbLAP1 is the first protein known to be localized within the nabelschnur. This morphologically prominent structure was first observed and defined by transmission electron microscopy under treatment with ethanolic phosphotungstic acid, a technique that highlights highly basic proteins [28]. The nabelschnur is formed by two parallel filaments that form a bridge between the segregating kDNA networks [28]. TbLAP1 has a basic pI of 9.8, which suggests the potential to interact with nucleic acids. It is noteworthy that the localization of TbLAP1 to the kDNA disk usually does not cover the entire network. This is particularly evident at the G_1_ phase and immediately before cytokinesis, when progeny kDNA networks have completed segregation and the TbLAP1 foci are asymmetric, yet invariably colocalize with the kDNA disk.

Cytosolic forms of LAP are present in most eukaryotes, but the expansion into three paralogs in *T*. *brucei*, revealed by phylogenetic analysis (S1 Fig, S2 Fig) is notable and suggests lineage-specific evolution of these proteins. The division of functions between LAPs following their expansion is apparently unique for trypanosomes and does not inform us about the functions of LAP paralogs in other lineages. In *T*. *cruzi* LAP has been described as a cytosolic protein displaying enzymatic activity [53] being orthologous to the protein studied here (S2 Fig). Down-regulation of TbLAP1 resulted in a growth defect, as well as in the accumulation of cell doublets. DAPI counts and PI staining of the TbLAP1-depleted cells suggests delayed cytokinesis. On the other hand, ectopic expression of TbLAP1-HA causes the loss of kDNA and concomitant growth defects. Since the process of kDNA segregation finishes with the onset of cytokinesis, both the effect of down-regulation and ectopic expression of TbLAP1 indicate its clear involvement in segregation of kDNAs. Although our results cannot clearly establish a molecular model for the process driven by TbLAP1, we propose that this protein is involved in the machinery that orchestrates and possibly times the final stages of cell division, led by the movement of the basal bodies. Similar phenotypes have been observed upon RNAi-mediated depletion of katanins and spastin in bloodstream form *T*. *brucei*, with these proteins known to be involved in cytokinesis [54]. However, the down-regulation of TbLAP1 does not seem to cause a defect in cell division, since as long as 8 days post RNAi-induction, no multinucleated cells were observed in the culture. While it may be argued that the tag impairs the function of the ectopic copy in a dominant-negative fashion, we have found that *in situ* tagging of TbLAP1, with either short V5 or long GFP tag does not produce the phenotype caused by the ectopic copy, though it exhibits exactly the same localization (S6 Fig).

Overexpression of a cytochrome b_5_ reductase–like protein renders kDNA incapable of duplication prior to cell division [55], whereas excess levels of two of six kDNA helicases induce a gradual loss of kDNA [27]. Ectopically expressed TbLAP1-HA accumulates around the kDNA disk, inducing a rapid loss of the structure. Several proteins are known to be important for kDNA segregation [56,57] or its maintenance [22,27,58,59], yet none of them display a localization similar to that of TbLAP1, nor a kDNA loss of this type.

**Fig 9:**
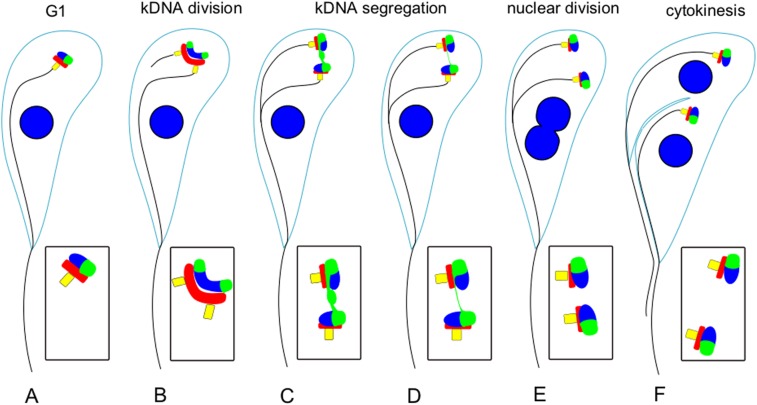
A model for the mutual relationships between the basal body, tripartite attachment complex and kDNA. Basal body (yellow), flagellum (black line), tripartite attachment complex (TAC) (red), kDNA and nucleus (blue), and TbLAP1 (green) are depicted during the cell cycle of procyclic *T*. *brucei*. (A) 1N1K interphase cells bearing one basal body, a flagellum and a single TAC connected with kDNA and TbLAP1. (B) Cells in the earliest stages of division, with basal and pro-basal bodies divided and started to segregate, and TAC division commenced; the growth of the new flagellum becomes evident; kDNA displays an elongated “dumbbell” shape, which reflects its replication; TbLAP1 co-localizes with kDNA. (C) Later stage where kDNA replication is complete and the progeny kDNA networks have divided and appear in a perpendicular position to one another; TbLAP1 is present in the progeny kDNAs and in the nabelschnur; basal and pro-basal bodies proceed with their segregation. (D) Later still, segregation of the newly divided kDNAs continues, together with respective TAC and basal bodies; the nabelschnur begins to fade. (E) Following nuclear DNA replication and segregation, newly divided nuclei appear, as the segregation of kDNA continues. (F) 2K2N cells just prior to cytokinesis, with nuclei completely divided with kDNA and associated structures aligned for the upcoming cell division.

Ectopically expressed TbLAP1 disrupted kDNA disk segregation, but division and segregation of the basal bodies were not affected. The basal body has been described to mediate kDNA segregation [60] and the same has been implied for TAC-associated proteins, such as p166 [56]. Moreover, it is well established that nuclear division and segregation are independent of the mechanism of kDNA segregation [47,61]. Throughout the cell cycle, the TbLAP1-HA signal dynamically changes, and its movement resembles that of the basal bodies (Fig 9). The stress experienced by trypanosomes expressing TbLAP1-HA is further reflected by the recruitment of mtHsp70 and its colocalization with TbLAP1-HA. Moreover, the viability of the newly divided cells was seriously hampered, as observed by the accumulation of 0K1N cells. It is likely that the collapse of mt membrane potential is a secondary effect of kDNA loss, as the same phenotype occurs following the depletion of several kDNA polymerases [20]. On the other hand, the down-regulation of TbLAP1 affects the separation of 2K2N cells but does not seem to affect division. These results strongly associate the protein with the segregation process and with the basal bodies as its orchestrators. Moreover, they provide further support for the independence of the division and segregation processes in the parasite.

In an attempt to determine proteins interacting with TbLAP1, we constructed a C-terminal *in situ* GFP-tagged version of the protein and the corresponding cell line was used to immunoprecipitate TbLAP1-GFP using anti-GFP nanobodies (S6 Fig). Although the eluate did not contain any other proteins than TbLAP1-GFP, we noticed that the protein was not able to enter the polyacrylamide gel. Reversion of this phenomenon upon DNAse treatment and/or sonication suggested binding to kDNA (S7 Fig)[53]. Multiple attempts and alternative immunoprecipitation conditions did not yield any TbLAP1-interacting proteins, although both the tagged and untagged proteins were efficiently pulled down, likely a consequence of its capacity to form oligomers, as described in other eukaryotes [65]. We explain this result as a consequence of the short-lived nature of the nabelschnur, which is observed only at the moment of kDNA segregation. Since kDNA replication commences before that of the nucleus and lasts for only a short period of time, at any point in a non-synchronized culture a very small percentage of cells are undergoing the G_2_/M phase transition, during which the newly synthesized kDNAs separate. While our experiments cannot assert that TbLAP1 binds kDNA directly, the association with the structure is evident.

Interaction of LAP with DNA has been observed in other organisms. DNA-associated PepA from *E*. *coli* regulates the pyrimidine-dependent repression of the carbamoylphosphate operon transcription [35] and participates in the site-specific Xer recombination system [62,63]. It is worth noting that proteolytic activity is not necessary for recombination activity [64]. Analysis of the TbLAP1 sequence indicates that not all of the amino acids involved in DNA binding and recombination activity of the *E*. *coli* orthologue are present in the *T*. *brucei* protein (S3 Fig, S4 Fig and S5 Fig). Of the nine amino acids required for exclusive binding of PepA to DNA [65], TbLAP1 contains only two (S3 Fig). Furthermore, TbLAP1 displays extra 100 amino acids that are absent in all the other LAPs assessed, and this sequence does not correspond to any known domain (S1 Fig, S3 Fig). In other organisms such as basidiomycetes, LAP promotes meiosis and may also be involved in DNA repair [36]. DNA binding of LAP has also been observed in human esophageal carcinoma, where it promotes G_1_/S transition, yet the underlying mechanism remains unknown [66]. The cysteinyl-glycyl activity is a more recently described function of LAPs. Although highly specialized when compared to that of the cleavage of N-terminal peptides, this LAP activity ranging from bacteria to mammals was proposed to recycle cysteinyl-glycyl in the γ-glutamyl cycle [42,67]. However, many components of both the γ-glutamyl cycle and the urea cycle with which it is closely associated, are absent from the *T*. *brucei* genome [68]. Hence, this enzymatic pathway likely does not take place in trypanosomatids, where glutathione is just one of several precursors for the subsequent formation of trypanothione. In summary, a new function and subcellular localization of LAP has been described in *T*. *brucei*, where the protein is involved in a unique mechanism.

## Materials and methods

### Phylogenetic reconstruction and sequence analysis

Dataset for phylogenetic analysis was created from publicly available sources, using BLASTP at an E-value cut-off of 1x10^-20^. The dataset was aligned by MUSCLE [69] and informative positions were selected using Gblocks [70] with manual adjustment in Seaview 4.6.1[71]. Maximum likelihood tree was constructed using RAxML 8.2.1 [72] with LG+GAMMA model and 1 000 bootstrap replicates. TriTrypDB and Genbank accession numbers for the sequences used are listed as follows: *Trypanosoma brucei* Tb927.8.3060 (TbLAP1), Tb927.11.6590 and Tb927.11.2470; *Trypanosoma cruzi* 1 EAN87580.1; *Trypanosoma cruzi* 2 EAN97960.1; *Trypanosoma cruzi 3* EAN99056.1; *Plasmodium falciparum*, XP_001348613.1; *Solanum lycopersicum* LAP-A gi|350540058|ref|NP_001233862.1|; *Solanum lycopersicum* LAP-N AAO15916.1; *Escherichia coli; Macaca mulatta* NP_001247627.1; *Homo sapiens* NP_056991.2; *Bos taurus* NP_776523.2; *Schizosaccharomyces pombe*, NP_592993.1; *Oryza sativa*, NP_001066684.1; *Mus musculus*, NP_077754.3; *Gallus*, NP_001026507.1; *Dictyostelium discoideum*, XP_641537.1; *Danio rerio*, *NP_001108319*.*1; Arabidopsis thaliana 3 NP_194821*.*1; Arabidopsis thaliana 2*, NP_194820.1; *Arabidopsis thaliana* 1, NP_179997.1.

### Construction of cell lines

A TbLAP1 RNAi cell line was constructed by cloning into the p2T7^Ti^-177 vector [73] a PCR amplified 400 bp-long region of the TbLAP1 gene using primers TTGGTGTTGAGCTTCTGGTG and TTGCCAGACCTTTTCTTTCC. The final construct was linearized with *Not*I and transfected into procyclic SMOXP9 *T*. *brucei* [74]. For overexpression, the full-size TbLAP1 gene, amplified with primers AAAAGTAAAATTCACGGGCCCATGCTCAAGAGAGT and CAGATTTTCGTTTCTGGTACCTCAATTGCCAGACCT (inserted *Apa*I and *Kpn*I restriction sites are underlined, respectively), was cloned into the *Hind*III+*Bam*HI pre-digested p2329 vector [75] using the Geneart Seamless Cloning and Assembly kit (Life Technologies).

TbLAP1 was *in situ* tagged using the long PCR approach, with either GFP or V5 tag attached to its C-terminus. For the GFP-tag, the pMOTag 3G vector was used to amplify a PCR product, which was transfected into procyclic stage of *T*. *brucei* 427 cells [76]. The following primers were used. For GFP tagging, primers 5’-GAAAAGAAGAGGGTGAAAAAGGCACCTGCGGCCAAGCAGGGTCGCCGGGCAGTGAAGGGGAACCCGAAAGGAAAGAAAAGGTCTGGCAATGGTACCGGGCCCCCCCTCGAG-3’ and 5’-AACCCCCAGTTGTGGGAACTTACGTGTCGAAAAACACCCCTTCCTCACAATACAGAAACGAGCGGCACGGTGGTTCCAATGTGGCGGCCGCTCTAGAACTAGTGGAT-3’ were used [76]. *In situ* tagging with C-terminal V5-tag was performed using the pPOTv4 vector, in which the yellow fluorescent protein (eYFP) was replaced by the V5 tag [77]. The following primers were used: 5’-AGTAATGCGCCACGGGGCTGCATGTAACCCTGTTGATGTCATTGAGAACTATCTGGAGGACAAACTCGATGAAATCGACATATGGGTGGGTACCGGGCCCCCCCTCGAG-3’ and 5’-CACATCCTGATGTGTTGCTTCTCGCCGCACCTAGCACGGTGAAGGCCGTGAGCATGTATGTGTAGTGCAGAAGAGTAAAGAGCGTTTTGGCGGCCGCTCTAGAACTAGTGGAT-3’.

### Cell cultivation and transfection

All experiments were conducted SDM79 medium. The procyclic 13–13 and SMOXP9 (which we refer to as SMOX) cell lines were used as parental lines for the ectopic expression of TbLAP1-HA (referred to as TbLAP1-HA throughout the paper) and RNAi against TbLAP1, respectively. *In situ* tagging of TbLAP1 was performed in procyclic 427 and SMOX cell lines. The SMOX cell line was cultivated in SDM79 with puromycin (0.5 μg/mL) for the stable expression of tetracycline (Tet) repressor and T7 polymerase. The 13–13 trypanosomes were grown with phleomycin (5 μg/mL) for the maintenance of stable expression of pHD13-13-encoded Tet repressor [78], and the SMOX cell line was cultivated in the presence of puromycin (0.5 μg/mL). Transfections were performed with 10 μg of linearized vector or PCR product and 2 x 10^7^ cells per transfection using a BTX electroporator. Clones were selected as described previously [79]. Induction of RNAi and overexpression was initiated by the addition of 1 μg/mL Tet to the cultures. Cell numbers were monitored using a Beckman Coulter Z2 counter. All growth curves were started with 2 x 10^6^ cells/mL and subcultured to the same cell number every 24 hrs.

### Immunofluorescence and DAPI staining

Ectopically expressed and *in situ* tagged TbLAP1 was followed by immunofluorescence assay as described elsewhere [80], with minor modifications. Expression was induced with 1 μg/mL Tet and samples were taken at several time points, with parental cell lines used as controls. Cells were fixed with 4% (w/v) paraformaldehyde in phosphate buffered saline (PBS), permeabilized with 0.2% (v/v) TX-100 in phosphate buffered saline (PBS) on microscopy slides and then probed with primary antibodies in PBS/gelatin. Polyclonal anti-TbLAP1 and anti-HA antibodies produced in rabbits (Sigma-Aldrich), and monoclonal anti-mtHsp70 antibody (kindly provided by Ken Stuart) were used at 1:1,000 dilution. Monoclonal anti-TAC102 and YL1/2 antibodies (kindly provided by Torsten Ochsenreiter and Keith Gull, respectively) were used at 1:2,500 and 1:500 dilutions, respectively. Rabbit anti-V5 antibody (Sigma-Aldrich) was used at a dilution of 1:8,000. As secondary antibodies, Alexa Fluor 488 anti-rabbit and Alexa Fluor 555 anti-mouse (Life Technologies) were used. DNA was visualized using ProLong Gold antifade reagent with DAPI (Life Technologies), and DAPI counts were performed as described previously [81]. Immunofluorescence analysis was performed using a Zeiss microscope Axioplan 2, equipped with an Olympus DP73 digital camera and detection was carried out with cellSens software (Olympus). Image analysis was performed using Magic Montage plugin for ImageJ [82] and FIJI [83]. All zoomed image sections are approximately 1μm x 1μm.

### Transmission electron microscopy of *in situ* tagged TbLAP1

Procyclic *T*. *brucei* expressing *in situ* tagged TbLAP1-V5 were fixed in 4% formaldehyde and 0.1% glutaraldehyde in 0.1 M 4-(2-hydroxyethyl)-1-piperazineethanesulfonic acid buffer (HEPES) for 1 h at room temperature. After washing in 10 mM glycine in HEPES, cell pellets were embedded into 10% (w/v) gelatin at 37°C, and left on a rotating wheel in 2.3 M sucrose at 4°C for 4 days, after which they were frozen by immersion in liquid nitrogen. Ultrathin cryosections were obtained using an ultramicrotome EM FCS equipped with UCT cryochamber (Leica Microsystems). Sections were transferred onto formvar-carbon-coated grids using a drop of 2.3 M sucrose/2% methyl cellulose (1:1). They were then washed in HEPES, blocked in a solution containing 5% low-fat milk, 10 mM glycine and 0.05% (v/v) Tween 20 for 1 h and incubated overnight with anti-V5-tag antibody (50 μg/ml; Invitrogen) in the blocking solution at 4°C. After a wash in 2.5% (w/v) low-fat milk, 5 mM glycine and 0.025% (v/v) Tween 20 in HEPES, sections were incubated for 1 h in goat anti-mouse IgG conjugated to 10 nm gold particles (Aurion), diluted 1:40 in the washing solution. Sections were then washed in HEPES, distilled water, contrasted and dried using 2% methyl cellulose with 3% aqueous uranyl acetate solution diluted at 9:1, and examined in either 80 kV JEOL 1010 or 200 kV 2100F transmission electron microscopes. Background labeling was tested by a negative control, in the absence of primary antibody, under the same conditions as those described above.

### Western blot analysis and FACS analysis

Cell lysates were prepared in NuPAGE LDS sample buffer (Invitrogen) using 5 x 10^6^ cells per lane separated on Bolt 4–12% Bis-Tris polyacrylamide gels (Invitrogen) and transferred to a Amersham Hybond P PVDF membrane (GE Healthcare), which was subsequently hybridized with monoclonal anti-GFP (Roche), polyclonal anti-HA (Sigma) or monoclonal anti-tubulin antibodies at 1:1,000, 1:2,000 and 1:10,000 dilutions, respectively. After hybridizing with an appropriate secondary antibody conjugated with horseradish peroxidase (Sigma), Clarity ECL substrate (Bio-Rad) was used to visualize the proteins. Band densitometry analysis was analyzed using ImageJ software [82].

FACS analysis was performed using a FACS Canto II flow cytometer (BD Biosciences). For mt membrane potential measurement, 5 x 10^6^ cells procyclic trypanosomes overexpressing TbLAP1 were incubated with MitoTracker Red CMXRos or TMRE (Tetramethylrhodamine) at 27°C for 20 min in SDM79, spun, and the pellet was resuspended in 1 mL PBS. Ten thousand events were measured per sample and each set of samples was measured at least three times in independent experiments [84]. Propidium iodide (PI) stained cells were prepared as described elsewhere [85]. Briefly, 2 x 10^7^ RNAi-induced cells were collected by centrifugation, washed with PBS, resuspended in 200 μL ice-cold 0.5% formaldehyde/PBS and incubated for 5 min on ice. Next, they were fixed with 2 mL of ice-cold 70% ethanol in vortex and allowed to stand 1 hr on ice. To stain cells with PI, the sample was centrifuged at 1500 x *g*, resuspended in 1 mL PBS and incubated with 50 μg PI and 200 μg of RNase A at 37°C for 1 hr. Data were analyzed using Flowing Software (Turku Centre for Biotechnology).

### Quantitative real-time PCR analysis

qRT-PCR analysis was performed as previously described [86]. RNA from 2 x 10^8^ *T*. *brucei* cells was isolated using TRI Reagent (Sigma-Aldrich) and cDNA from 1 μg of total RNA was synthesized using Quantitect Reverse Transcription kit (Qiagen). TbLAP1 RT-qPCR was performed under non-saturating conditions, in triplicate using primers 5’-ATGTGGATAAACACGACGCA-3’ and 5’-GCTCCGGATCCAACAAAATA-3’ for TbLAP1 on a Roche LightCycler 480 (v1.5). Analysis of the data was performed using the Pfaffl method for relative quantification [87] after adjustment of primer efficiency, on 18S rRNA as a standard [88] with LinRegPCR software [89].

## Supporting information

S1 Fig Sequence alignment of TbLAPs. Alignment of TbLAP1 (Tb927.8.3060), TbLAP2 (Tb927.11.2470) and TbLAP3 (Tb927.11.6490) with LAP sequences of *Homo sapiens*, *Bos taurus*, *Arabidopsis thaliana*, *Solanum lycopersicum*, *Treponema denticola* and *Escherichia coli* PepA display the conserved motifs of the M17 family (squared) and metal binding sites (* and squares) present in all the members of the family.
(PNG)

S2 Fig Phylogenetic analysis of LAPs. Alignment of 21 sequences was performed using MUSCLE and edited using Seaview (sequence accession numbers in Materials and Methods). *E*. *coli* PepA was used as outgroup.
(TIF)

S3 Fig Sequence alignment of TbLAP1, LAPTc and *Escherichia coli* PepA exhibiting DNA-binding sites. Alignment was performed using Clustal Omega and edited with Geneious 9.1.5. All amino acids in red are required for binding of PepA to DNA. Asterisks indicate those amino acids that are present in TbLAP1. None of the required amino acids are present in LAPTc.
(TIF)

S4 Fig Sequence alignment of maxicircles, ColE1 and the *cer* site in ColE1, the binding site of PepA during Xer recombination. Alignment was performed using Clustal Omega and edited with Geneious 9.1.5. *cer* sequence protected by PePA was obtained from [63]. *T*. *brucei* maxicircle: M94286.1. ColE1: NC_001371.1
(TIF)

S5 Fig Sequence alignment of minicircles, ColE1 and the *cer* site in ColE1, the binding site of PepA during Xer recombination. Alignment was performed using Clustal Omega and edited with Geneious 9.1.5. *cer* sequence was obtained from [63]. *T*. *brucei* minicircles sequences were retrieved from Genbank: TBREP2, V01389.1; V01388.1; TRBCSGRA, L25588.1; TRBCSGRB, L25589.1; TRBINVRPTB, L16536.1; TRBINVRPTC, L16537.1; TRBINVRPTD, L16538.1; TRBINVRPTE, L16539.1; TRBINVRPTF; L16540.1; TRBINVRPTG, L16541.1; TRBINVRPTH, L16542.1; TRBINVRPTI, L16543.1; TRBKPGUIDE, L11652.1.
(TIF)

S6 Fig Immunofluorescence microscopy of *in situ* tagged TbLAP1-GFP. A) PCF427 control immunodecorated with monoclonal anti-GFP. B-E) Representative cells expressing TbLAP1-GFP, immunodecorated with monoclonal anti-GFP antibody. White arrowheads indicate the merge of the kinetoplast with the GFP signal, which is not observed in the WT (PCF427 shown in A). Panel E also shows evidence of nabelschnur formation.
(TIF)

S7 Fig Immunoprecipitation of TbLAP1-GFP using anti-GFP nanobodies. Approximately 10^10^ procyclic trypanosomes harboring TbLAP1-GFP were broken by mechanical milling in a Planetary Ball Mill PM200 using liquid nitrogen cooling (Retsch). Ten mg of broken cell material was used to immunoisolate the tagged protein through Dynabeads M-270 epoxy coupled to GFP nanobody. 427 *T*. *brucei* were used in the same fashion as control. Samples were dissolved in 1 mL of 20 mM HEPES (pH 7.4), 150 mM Na-citrate, 1.0 mM MgCl_2_, and 0.1 mM CaCl_2_ with three pulses of sonication of 5 sec each at 60% power, in the presence of protease inhibitors cocktail (Roche) and detergent. Beads were washed 5X and the protein was eluted using SDS-sample buffer in the absence of dithiothreitol at 72°C for 20 min. Western blot analysis (WB) and silver stained SDS-PAGE (SiS) of immunoprecipitated TbLAP1-GFP in presence of 0.1% (v/v) TX-100 and 0.1% (w/v) CHAPS. Immunoprecipitation controls with PCF427 whole cell lysates (SiS-C). *DNase samples were prepared without prior sonication and were treated with 30U DNase I (Qiagen) on beads for 30 minutes prior to washing. Only the samples in TX-100 showed an effect after DNase treatment and are shown in a western blot with anti-GFP antibody labeled as (-) and (+) DNase.
(TIF)

S8 Fig Immunofluorescence microscopy of induced TbLAP1-HA using MitoTracker. MitoTracker (red) was used to visualize the mitochondrion and monoclonal anti-HA antibody (green) detected TbLAP1-HA. DAPI (blue) stained DNA. It should be noted that MitoTracker accumulates in “patches” throughout the reticulated mitochondrion.
(TIF)

S1 Table Distribution of immuno-gold particles on kDNA. Percentages of clustered and randomly distributed immuno-gold particles on transmission electron microscopy images of kDNAs. Clustering of particles was determined by Ripley’s function.
(DOCX)
