# Supplementary figures and images for: A leucine aminopeptidase is involved in kinetoplast DNA segregation in Trypanosoma brucei
Source: PLoS Pathog. 2017 Apr 7;13(4):e1006310. doi: 10.1371/journal.ppat.1006310 (PMC5397073; doi:10.1371/journal.ppat.1006310)

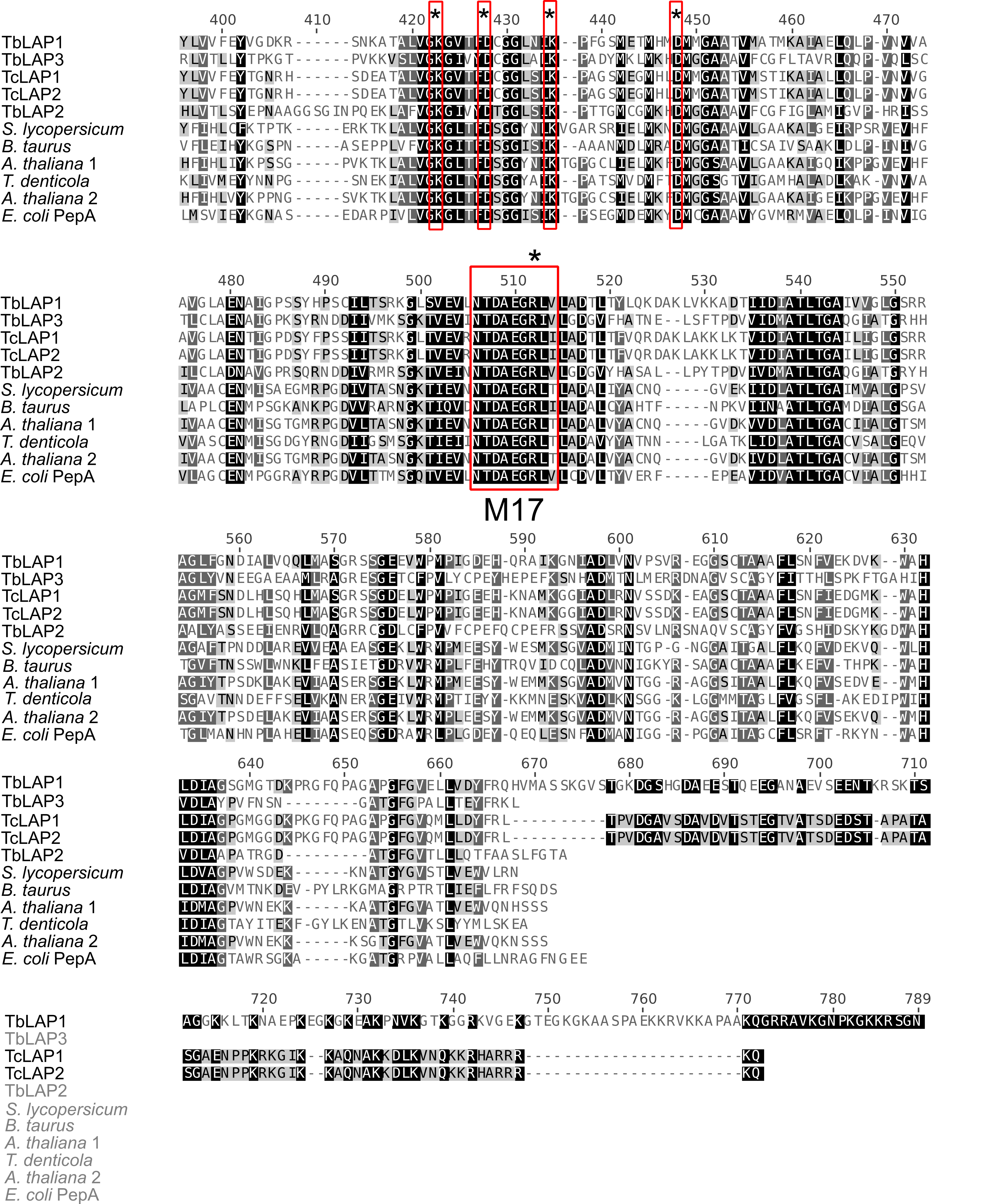

Supplement: S1 Fig — Alignment of TbLAP1 (Tb927.8.3060), TbLAP2 (Tb927.11.2470) and TbLAP3 (Tb927.11.6490) with LAP sequences of Homo sapiens, Bos taurus, Arabidopsis thaliana, Solanum lycopersicum, Treponema denticola and Escherichia coli PepA display the conserved motifs of the M17 family (squared) and metal binding sites (* and squares) present in all the members of the family. (PNG) [file ppat.1006310.s001.png]

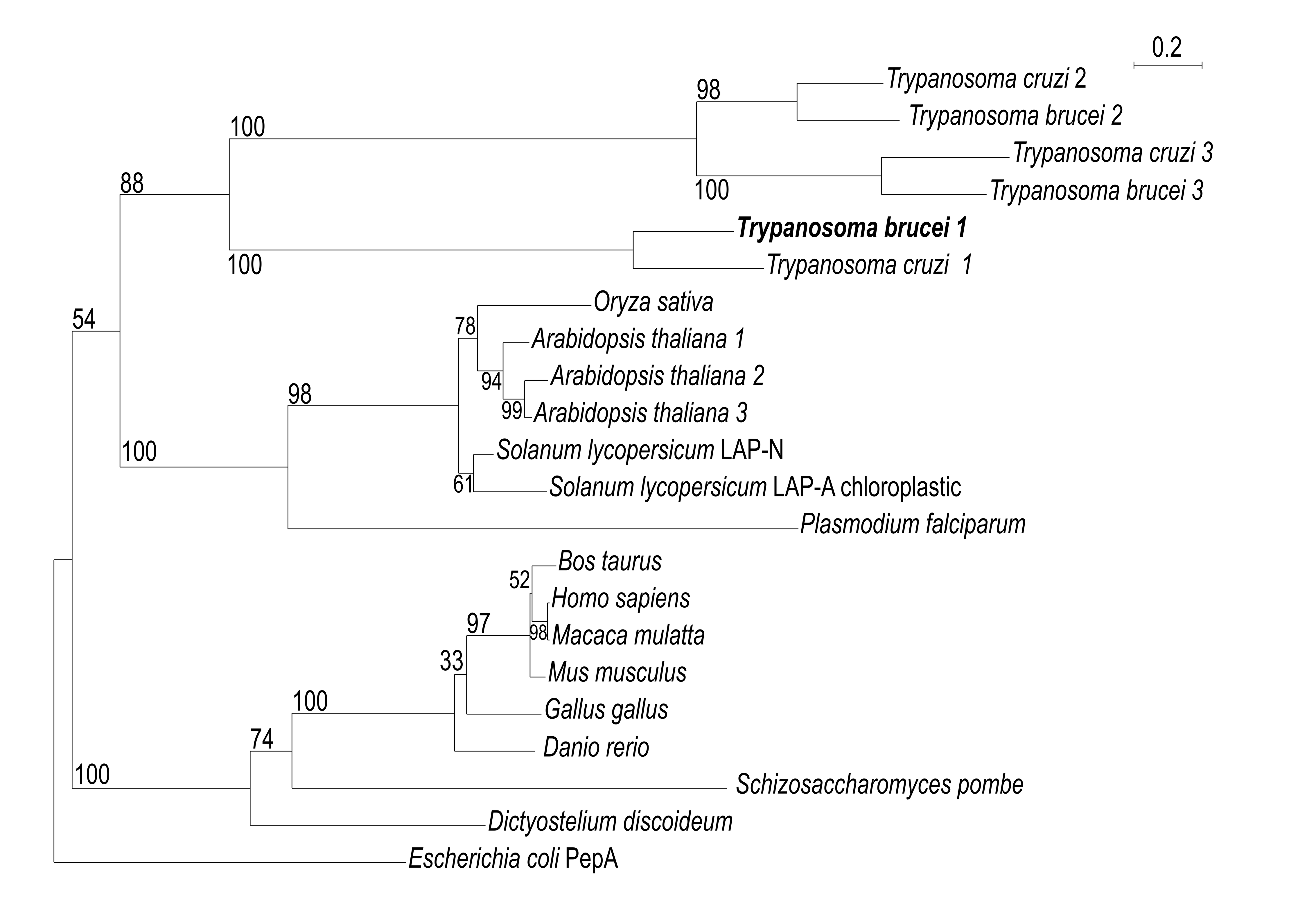

Supplement: S2 Fig — Alignment of 21 sequences was performed using MUSCLE and edited using Seaview (sequence accession numbers in Materials and Methods). E. coli PepA was used as outgroup. (TIF) [file ppat.1006310.s002.tif]

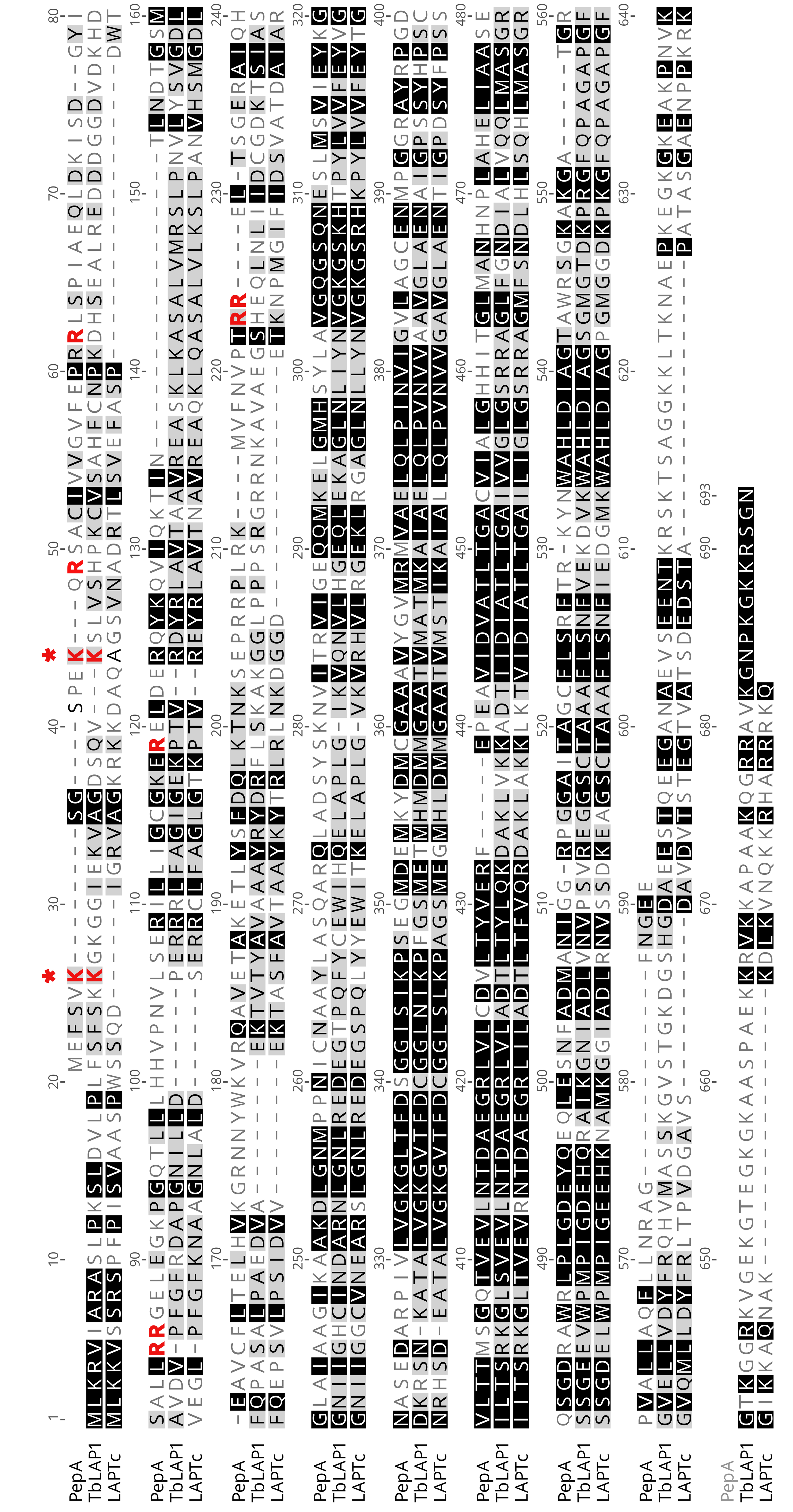

Supplement: S3 Fig — Alignment was performed using Clustal Omega and edited with Geneious 9.1.5. All amino acids in red are required for binding of PepA to DNA. Asterisks indicate those amino acids that are present in TbLAP1. None of the required amino acids are present in LAPTc. (TIF) [file ppat.1006310.s003.tif]

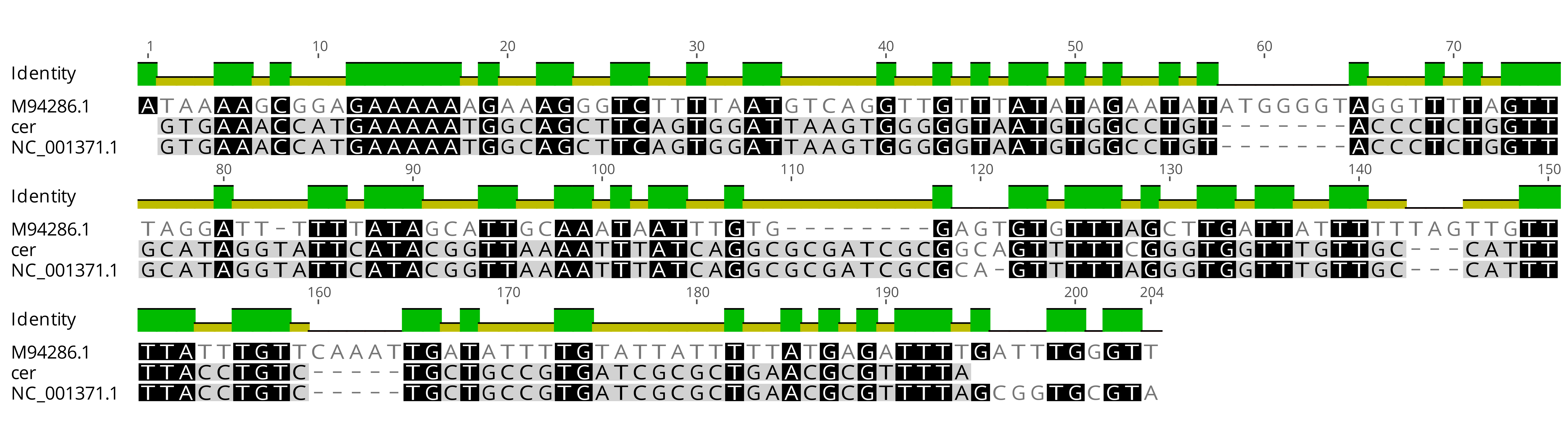

Supplement: S4 Fig — Alignment was performed using Clustal Omega and edited with Geneious 9.1.5. cer sequence protected by PePA was obtained from [63]. T. brucei maxicircle: M94286.1. ColE1: NC_001371.1 (TIF) [file ppat.1006310.s004.tif]

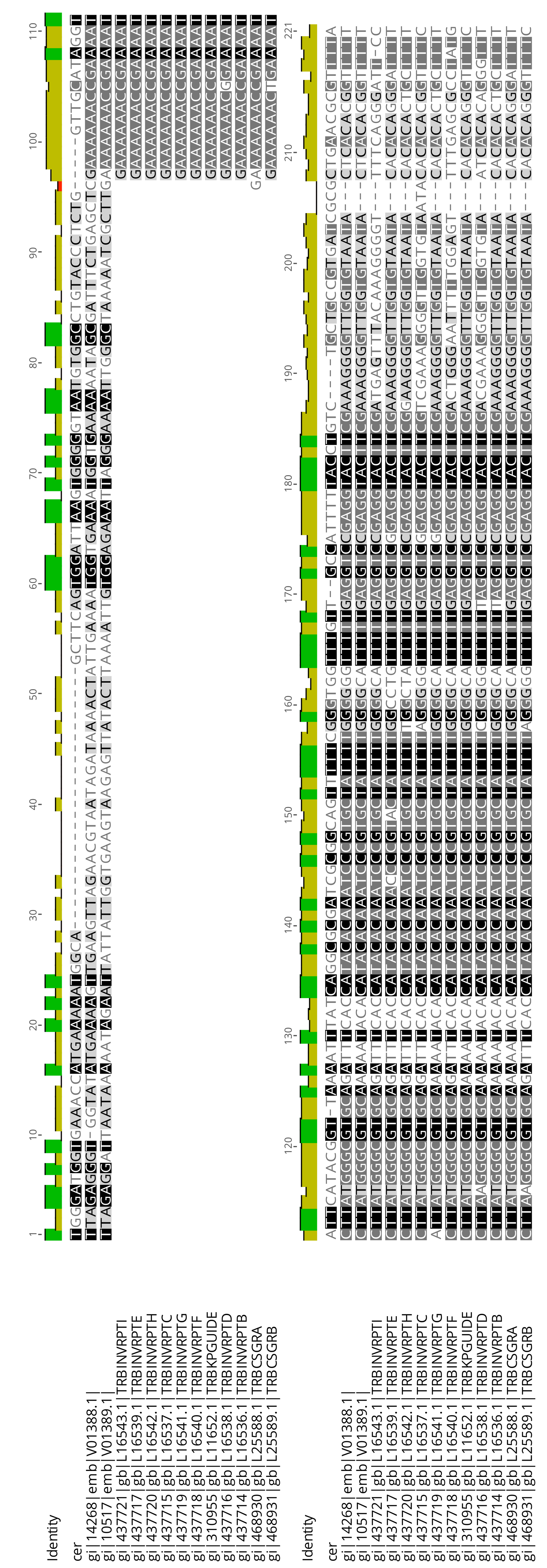

Supplement: S5 Fig — Alignment was performed using Clustal Omega and edited with Geneious 9.1.5. cer sequence was obtained from [63]. T. brucei minicircles sequences were retrieved from Genbank: TBREP2, V01389.1; V01388.1; TRBCSGRA, L25588.1; TRBCSGRB, L25589.1; TRBINVRPTB, L16536.1; TRBINVRPTC, L16537.1; TRBINVRPTD, L16538.1; TRBINVRPTE, L16539.1; TRBINVRPTF; L16540.1; TRBINVRPTG, L16541.1; TRBINVRPTH, L16542.1; TRBINVRPTI, L16543.1; TRBKPGUIDE, L11652.1. (TIF) [file ppat.1006310.s005.tif]

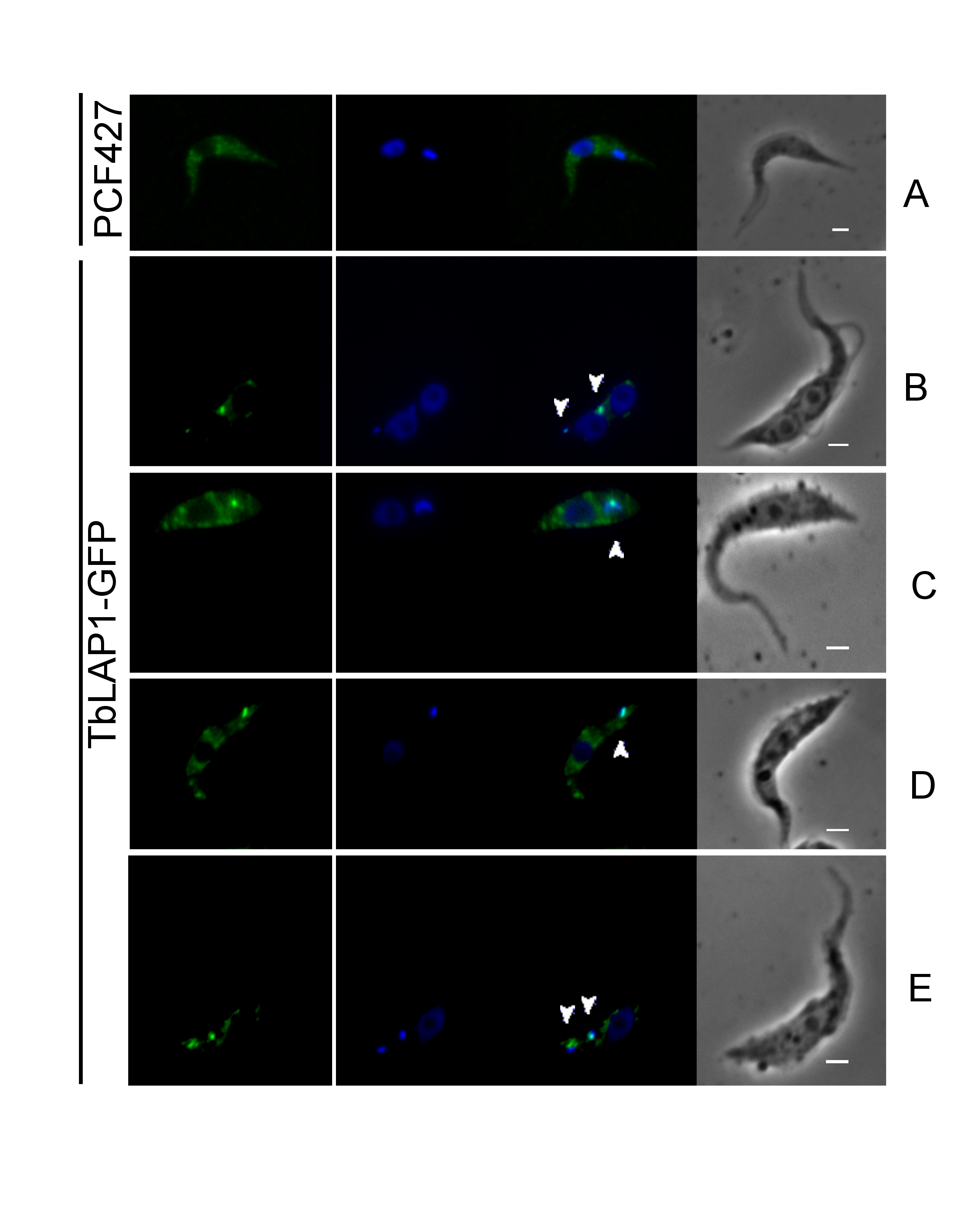

Supplement: S6 Fig — A) PCF427 control immunodecorated with monoclonal anti-GFP. B-E) Representative cells expressing TbLAP1-GFP, immunodecorated with monoclonal anti-GFP antibody. White arrowheads indicate the merge of the kinetoplast with the GFP signal, which is not observed in the WT (PCF427 shown in A). Panel E also shows evidence of nabelschnur formation. (TIF) [file ppat.1006310.s006.tif]

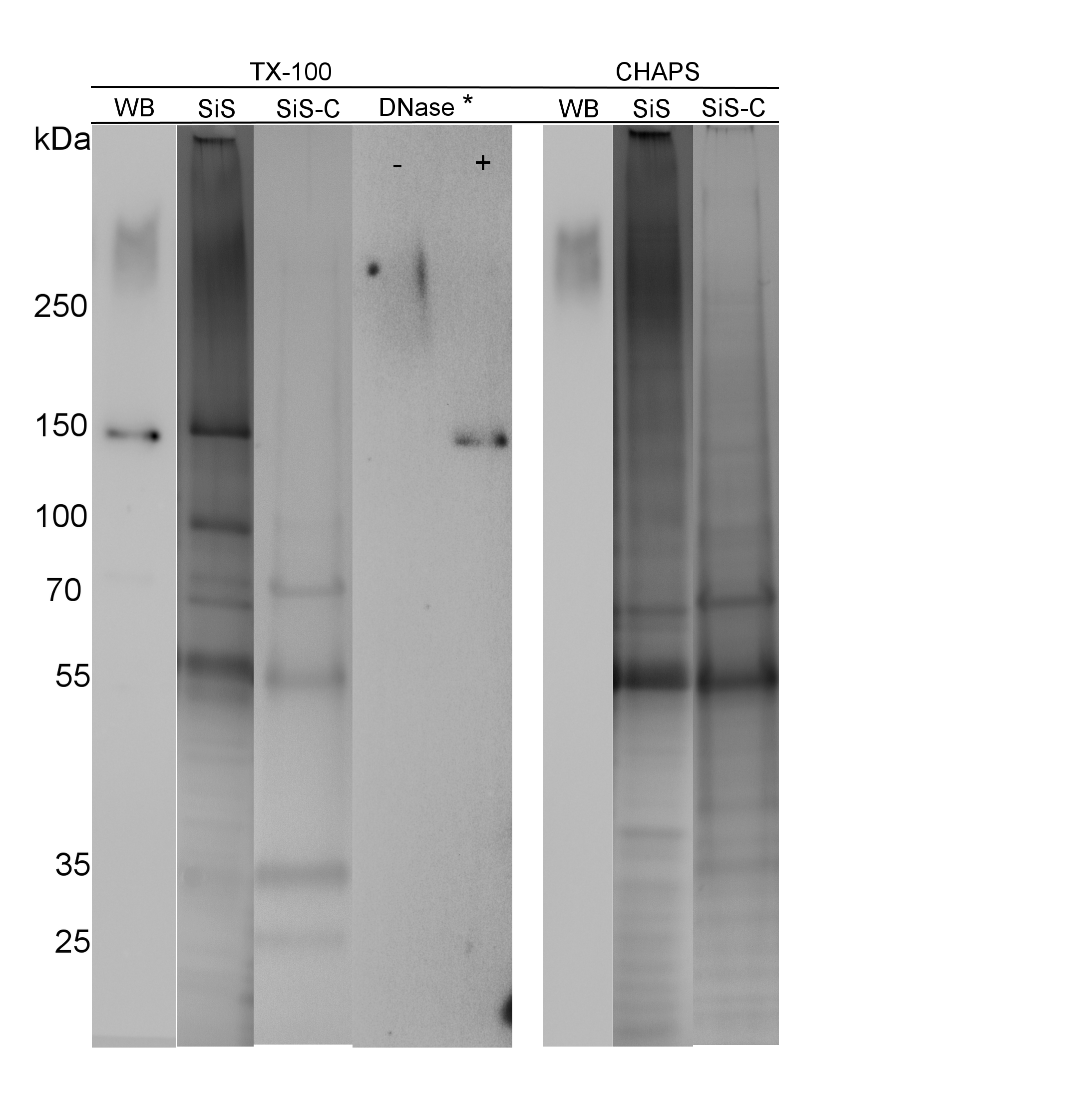

Supplement: S7 Fig — Approximately 1010 procyclic trypanosomes harboring TbLAP1-GFP were broken by mechanical milling in a Planetary Ball Mill PM200 using liquid nitrogen cooling (Retsch). Ten mg of broken cell material was used to immunoisolate the tagged protein through Dynabeads M-270 epoxy coupled to GFP nanobody. 427 T. brucei were used in the same fashion as control. Samples were dissolved in 1 mL of 20 mM HEPES (pH 7.4), 150 mM Na-citrate, 1.0 mM MgCl2, and 0.1 mM CaCl2 with three pulses of sonication of 5 sec each at 60% power, in the presence of protease inhibitors cocktail (Roche) and detergent. Beads were washed 5X and the protein was eluted using SDS-sample buffer in the absence of dithiothreitol at 72°C for 20 min. Western blot analysis (WB) and silver stained SDS-PAGE (SiS) of immunoprecipitated TbLAP1-GFP in presence of 0.1% (v/v) TX-100 and 0.1% (w/v) CHAPS. Immunoprecipitation controls with PCF427 whole cell lysates (SiS-C). *DNase samples were prepared without prior sonication and were treated with 30U DNase I (Qiagen) on beads for 30 minutes prior to washing. Only the samples in TX-100 showed an effect after DNase treatment and are shown in a western blot with anti-GFP antibody labeled as (-) and (+) DNase. (TIF) [file ppat.1006310.s007.tif]

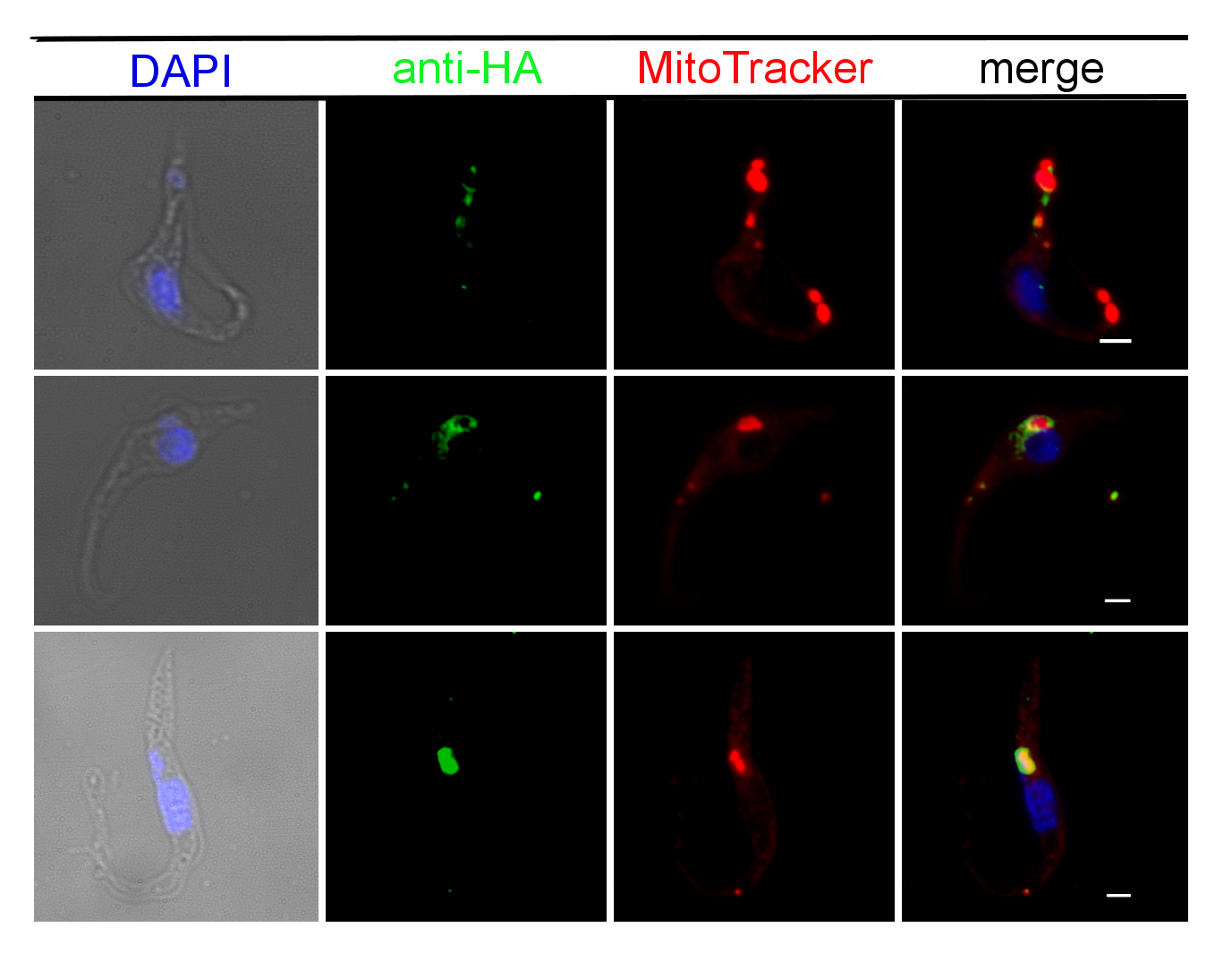

Supplement: S8 Fig — MitoTracker (red) was used to visualize the mitochondrion and monoclonal anti-HA antibody (green) detected TbLAP1-HA. DAPI (blue) stained DNA. It should be noted that MitoTracker accumulates in “patches” throughout the reticulated mitochondrion. (TIF) [file ppat.1006310.s008.tif]
