# Supplementary material for: A leucine aminopeptidase is involved in kinetoplast DNA segregation in Trypanosoma brucei
Source: PLoS Pathog. 2017 Apr 7;13(4):e1006310. doi: 10.1371/journal.ppat.1006310 (PMC5397073; doi:10.1371/journal.ppat.1006310)
Supplement: S1 Table — Percentages of clustered and randomly distributed immuno-gold particles on transmission electron microscopy images of kDNAs. Clustering of particles was determined by Ripley’s function. (DOCX) [file ppat.1006310.s009.docx]

**A leucine aminopeptidase is involved in kinetoplast DNA segregation in *Trypanosoma brucei***

Priscila Peña-Diaz^1,#^, Marie Vancová^1,2^, Christian Resl^2,*^, Mark C. Field^3^ and Julius Lukeš^1,2,4,#^

**Supplementary information**

**S1 Table. Distribution of immuno-gold particles on kDNA.**

| Fig. | N gold particles | Clustering (Y/N) |
| --- | --- | --- |
| 1 | 10 | N |
| 2 | 12 | N |
| 3 | 26 | N |
| 4 | 11 | Y |
| 5 | 7 | N |
| 6 | 51 | Y |
| 7 | 22 | N |
| 8 | 24 | Y |
| 9 | 22 | Y |
| 10 | 15 | Y |
| 11 | 34 | N |
| 12 | 13 | N |
| 13 | 13 | Y |
| 14 | 14 | N |
| 15 | 10 | N |
| 16 | 6 | Y |
| 17 | 10 | N |
| 18 | 18 | N |
| 19 | 11 | N |
| 20 | 13 | Y |
| **20** | **342** | **40%Y** |
